# Long-term survival and clinical outcomes of non-vascularized autologous and allogeneic fibular grafts are comparable for treating osteonecrosis of the femoral head

**DOI:** 10.1186/s13018-021-02246-3

**Published:** 2021-02-04

**Authors:** Ke Jie, Wenjun Feng, Feilong Li, Keliang Wu, Jinlun Chen, Guanming Zhou, Huiliang Zeng, Yirong Zeng

**Affiliations:** 1 Foshan Hospital of Traditional Chinese Medicine, Foshan, 528000 Guangdong Province China; 2 The Third Department of Orthopedics, The First Affiliated Hospital of Guangzhou University of Chinese Medicine, 16 Jichang Road, Baiyun District, Guangzhou, 510405 Guangdong Province China; 3 The Third Affiliated Hospital of Guangzhou University of Chinese Medicine, Jiangnanxi Road, Haizhu District, Guangzhou, 510405 Guangdong Province China; 4 School of Shenzhen Bao’An Shajing People’s Hospital, Guangzhou University of Chinese Medicine, 12 Jichang Road, Baiyun District, Guangzhou, 510405 Guangdong Province China

**Keywords:** Osteonecrosis of the femoral head, Hip-preserving surgery, Autologous fibular grafting, Allogeneic fibular grafting

## Abstract

**Background:**

Osteonecrosis of the femoral head (ONFH) is a disabling disease, which often involves young patients. Recently, various hip-preserving surgeries were recommended to delay total hip arthroplasty (THA).

**Questions/purposes:**

This study aimed to compare clinical outcomes and survival rate in the long-term follow-up between core decompression combined with a non-vascularized autologous fibular graft (group A) and an allogeneic fibular graft (group B) for the treatment of ONFH.

**Patients and methods:**

We retrospectively evaluated 117 patients (153 hips) with ONFH (Association Research Circulation Osseous [ARCO] stages IIa to IIIc) who underwent the abovementioned hip-preserving surgeries between January 2003 and June 2012. The mean (range) follow-up times (years) were 12.9 (7–16) and 9.3 (6–16) in groups A and B, respectively. Clinical outcomes were assessed using the Harris Hip Score (HHS), visual analog scale (VAS) score, and forgotten joint score (FJS). A survival analysis was performed using the Kaplan-Meier method. The end point was THA.

**Results:**

Groups A and B showed postoperative improvements, respectively, in HHS from 65 ± 7.2 to 80.3 ± 14.5 and from 66 ± 5.9 to 82.4 ± 13.6 (*p* < 0.05), and in VAS score from 6.3 ± 1.1 to 2.3 ± 1.6 and from 6.1 ± 1 to 2.2 ± 2.2 (*p* < 0.05). However, no significant differences in the HHS, VAS score, and hip FJS at the last follow-up (*p* > 0.05) and 15-year survival rate (84.1% and 86%, respectively, *p* > 0.05) were found between groups A and B.

**Conclusions:**

Autologous and allogeneic fibular grafts can attain equally good clinical outcomes and high survival rates in long-term follow-up, and thus can greatly delay THA owing to good bone osseointegration and sufficient mechanical support. Notably, the ratio of failure will increase when patients were more than 37 years old.

**Level of evidence:**

Level III, therapeutic study

**Supplementary Information:**

The online version contains supplementary material available at 10.1186/s13018-021-02246-3.

## Background

Osteonecrosis of the femoral head (ONFH) is a disabling disease with the process of destruction of the femoral head, bone cell degeneration and necrosis, subchondral bone collapse, and final articular cartilage degeneration and osteoarthritis [1]. The peak age of onset of ONFH was reported in young patients, especially in men in their 40s and women in their 30s, who have reached the pinnacle of their career and physical development [2].

Total hip arthroplasty (THA) is a preferable surgical option for the treatment of middle-to-late-stage ONFH [3]. However, young and middle-aged patients will undergo one or more revisions due to prosthesis wear and loosening [4, 5]. Hip-preserving surgeries have been proved in many studies to be effective for promoting blood supply reconstruction, repairing bone tissue, providing mechanical support, and preventing femoral head collapse [6–8]. Thus, they have a broad application prospect in the treatment of young and middle-aged patients with ONFH, including core decompression (CD), vascularized bone grafting, free vascularized bone grafting, vascularized greater trochanter flap, porous tantalum rod implant, transtrochanteric rotational osteotomy, etc. [9–12].

CD alone is a minimally invasive procedure for the treatment of early-stage ONFH, but the outcome is unsatisfactory owing to the lack of structural support for the subchondral plate [13, 14]. To prevent and treat femoral head collapse, the porous tantalum rod is implanted after CD to provide enough mechanical support [9]. However, studies have shown that bone osseointegration in the porous tantalum rod was inferior [15], and the survival rates in the mid-term, long-term, and even the early follow-up periods were low [16, 17]. Transtrochanteric rotational osteotomy is performed to transform a necrotic area into a non-weight-bearing area, which can reduce the intraosseous pressure and provide a good repair environment [18]. Nevertheless, this procedure also has limitations. The operation is complicated, traumatic, and easily damages peripheral blood vessels such as the medial circumflex femoral artery [19]. Therefore, its popularity has gradually diminished. Vascularized autologous fibular graft is an another method for the management of ONFH [20]. Although it can not only increase the blood supply in the femoral head but also provide mechanical support, it is limited due to the high surgical technique, separation of arteries, complications in donor area, and uncertain efficacy [21–23].

Compared with other hip-preserving surgeries, CD combined with fibular grafting not only provides sufficient mechanical support but also possesses better bone osseointegration [9, 22, 24]. The shape of the fibula is straight and long, and its cylindrical structure can be in maximum contact with the surrounding bone and match the subchondral bone well. The fibular graft can be divided into autologous and allogeneic sources. In theory, the autologous fibula is considered superior to the allogeneic fibula for the following reasons: (1) As for the autologous bone grafting, immunologic rejection can be avoided. (2) Autologous bone grafting promotes a stronger healing ability, which can better increase the mechanical support of the subcartilage bone in the load-bearing area of the femoral head and promote the repair of necrotic bone. (3) It can also reduce patients’ surgical expenses. However, the autofibular bone source is limited, and various donor site morbidities can occur [24, 25]. It was reported that CD combined with allogeneic bone grafting can also achieve satisfactory results [26]. To our knowledge, a unified consensus still lacks on the curative effect and survival rate of autologous and allogeneic fibular grafting for managing ONFH, especially in the mid- and long-term follow-up.

We hypothesized that the clinical outcomes of non-vascularized allogeneic fibular grafting were similar to those of non-vascularized autologous fibular grafting. Moreover, the treatment of early-to-middle-stage ONFH with allogeneic fibular grafting can reduce not only the injury but also the unnecessary discomfort in the donor area. Therefore, the purpose of the present study was to evaluate the therapeutic effects and survival rates of the 2 types of fibular grafting techniques, which can provide clinical evidence for choosing the surgical plan for early-to-middle-stage ONFH.

## Methods

This study was approved by the Ethics Committee of the First Affiliated Hospital of Guangzhou University of Traditional Chinese Medicine and conducted in accordance with the principles of the Declaration of Helsinki. All patients undergoing core decompression with addition of a fibular graft (allogeneic or autologous) for ONFH by the same senior surgeon at the First Affiliated Hospital of Guangzhou University of Traditional Chinese Medicine between January 2003 and June 2012 with a minimum 6-year follow-up were selected. The diagnosis of ONFH was based on history, symptoms, signs, and radiography, computed tomography (CT), and magnetic resonance (MR) findings. The indications for this procedure were as follows: (1) patients with symptomatic ONFH of ARCO stage II or III, (2) those who could the follow postoperative training programs, and (3) those aged between 18 and 60 years. Patients were not considered candidates for the procedure if they are (1) those with a previous history of hip surgery, (2) those with a previous history of hip infection, (3) those with hip deformities, and (4) those who should continue to be treated with high-dose corticosteroid therapy after operation.

After screening, 131 patients (173 hips) were included in the present study. Among the patients, 14 were excluded for the following reasons: (1) 10 patients (16 hips) did not cooperate during follow-up; (2) 3 patients (3 hips) needed further oral administration of large amounts of glucocorticoids for systemic lupus erythematosus; (3) 1 patient (1 hip) was considered as having ARCO stage IV ONFH, who was eager for hip-preserving surgery.

The remaining 117 patients (153 hips) were finally enrolled (follow-up rate, 89.3%), including 96 males and 21 females, with a mean age of 37.2 years (range, 18–59 years), and mean body mass index (BMI) of 24.2 ± 2.7 kg/m^2^. Patients who underwent CD with autologous and allogeneic fibular grafting were assigned to groups A and B, respectively. The autologous and allogeneic techniques had been performed since 2003, and after 2007, only allogeneic technique was performed. The mean follow-up time was 12.9 ± 2.8 years (range, 7–16 years) in group A and 9.3 ± 3 years (range for the patients who did not undergo conversion surgery to THA, 6–16 years) in group B. No significant differences in sex, BMI, etiological composition, and ARCO stage were found between the 2 groups (*p* > 0.05). The patients in group A were older (*p* < 0.05) and had longer follow-up periods (*p* < 0.05) than those in group B. The operation duration, material cost, and intraoperative blood loss were also recorded. More details are presented in Table 1. All baseline data were preoperative.

**Table 1:** Comparison of baseline characteristics of group A and group B

Baseline characteristics	Group A	Group B	*p* value
No. of patients (hips)	34(50)	83(103)	
Age (years; mean ± SD, [range])	41.6 ± 8 (23 to 58)	35.7 ± 10.6 (18 to 59)	0.0108
Sex (female/male)	30/4	66/17	0.303
Follow-up (years; mean ± SD, range)	12.9 ± 2.8 (7 to 16)	9.3 ± 3 (7 to 16)	< 0.001
BMI (median, mean ± SD)	23.8 ± 2.9	24.4 ± 2.6	0.2755
Operation duration (min, mean ± SD)	102.3 ± 18.3	83 ± 19.5	< 0.0001
Material cost (renminbi, mean ± SD)	1080 ± 1587.1	13707.8 ± 3779.5	< 0.0001
Intraoperative blood loss (ml, mean ± SD)	169 ± 78	129.5 ± 67.2	0.0015
Etiologies (%)			0.09
Glucocorticoid	12 (35.3%)	31 (37.3%)	
Alcoholic	19 (55.9%)	29 (34.9%)	
Traumatic	2 (5.9%)	16 (19.3%)	
Idiopathic	1 (2.9%)	7 (8.4%)	
Pre-ARCO stage (*n*, %)			0.977
IIA	5 (10%)	9 (8.7%)	
IIB	12 (24%)	21 (20.4%)	
IIC	15 (30%)	37 (35.9%)	
IIIA	8 (16%)	15 (14.6%)	
IIIB	5 (10%)	9 (8.7%)	
IIIC	5 (10%)	12 (11.7%)	

Note: *BMI* body mass index, *Pre-ARCO stage* preoperative Association Research Circulation Osseous stage, *SD* standard deviation

### Surgical technique

**Fig. 1:**
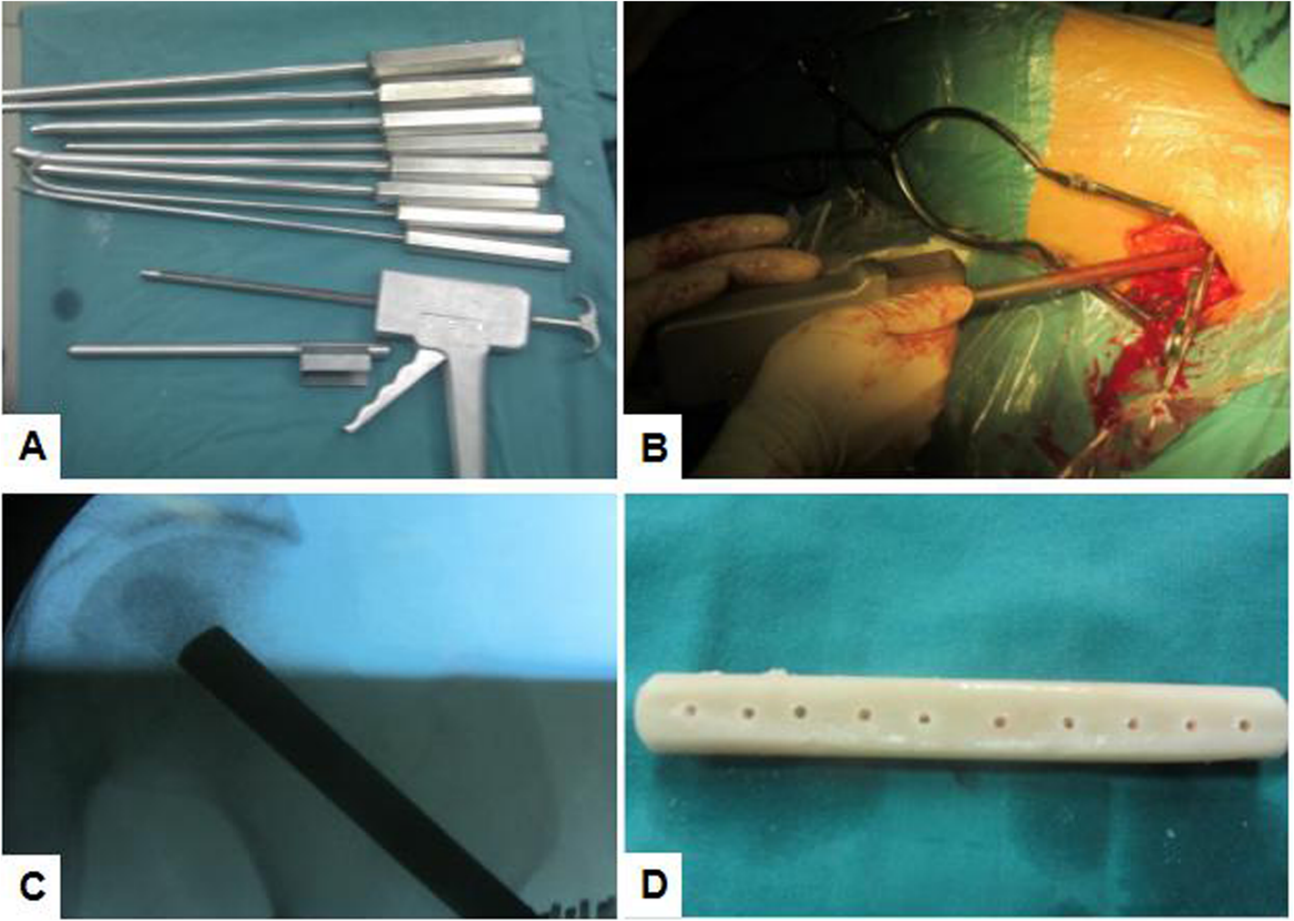
**a** A photograph of straight and curved impaction rods in different sizes and a bone impaction instrument. **b** A surgical procedure of filling cancellous bone layer by layer. **c** A radiograph of cancellous bone impaction grafting. **d** A photograph of the allogeneic fibula after drilling holes for the purpose of enhancing bone ingrowth

The original surgical technique was previously described in detail [27] and partly modified by us. For group B, the detailed steps were mainly divided into 3 parts as follows: localization, establishment of a bone canal, and fibular grafting. First, the patient was placed in a supine position, with a towel under the buttock to raise the femoral head. A 5-cm-long longitudinal skin incision was made downward along the greater trochanter for a lateral approach to the hip. Under the C-arm monitoring, a Kirschner wire was drilled into the femoral head to reach the necrotic area of the femoral head. Then, CD was performed up to 5 mm below the cartilage of the femoral head to eliminate the necrotic bone effectively. In the second step, 8 mm- to 12 mm-sized “T” shape hand drillers were used manually to expand the bone tunnel. In the final step, hole was drilled in the fibula with 3.0-mm Kirschner wire at the intervals of about 1 cm to increase bone growth, which was from the surface of one side to the opposite side throughout the medullary cavity of fibula, and the top of fibula was reshaped with an osteotribe to enhance matching. A pressurizer was used for allogeneic bone granule grafting to fill up the cavity tightly, followed by fibular grafting along the bone canal. During the grafting, cancellous bone was grafted layer by layer and tightly impacted in all directions. As described by Penix et al [28], the fibula was placed as close as possible in the lateral part of head. For group A, the procedure used was the same as that for group B, except for the non-vascularized autologous fibular grafting. The osteotomy length was approximately 75% of the upper section of the fibula. Several procedures are showed in Fig. 1.

After surgery, negative pressure drainage and intravenous antibiotic prophylaxis were performed for 1 day. Joint function exercise and muscle strength recovery was started on the second postoperative day. All the patients were hospitalized for around 1 week and maintained non-weight bearing for the first 6 weeks postoperatively, followed by partial weight bearing according to the bone necrotic area repair in the outpatient review. The patients were asked to maintain lower extremity skin traction 12 h a day within half a year, gradually reducing it. Total weight bearing was usually permitted in the sixth postoperative month.

### Outcome evaluation

The patients were reviewed at outpatient clinic and were also regularly contacted by telephone and postal questionnaire at 1, 3, 6, and 9 months within 1 year after surgery and yearly thereafter. The Harris Hip Score (HHS), visual analog scale (VAS) score, forgotten joint score (FJS), and patient satisfaction level were used for the clinical assessment. VAS was about the pain of hip with the total scores of 10. 0 score means no pain and a higher score means a higher level of pain. The clinical outcomes obtained face-to-face.

The FJS was introduced by Behrend et al. in 2012 [29]. It has been proved effective, reliable, feasible, and responsive, with a small “ceiling effect,” which has significant advantages in subjective feelings. A higher score means a higher level of forgetting the surgical joint and a lower level of feelings. The FJS was only recorded at the last follow-up.

Patient satisfaction was divided into 4 levels as follows: strongly satisfactory, moderately satisfactory, unsatisfactory, and poor. Satisfaction was defined when the patient answered “strongly satisfactory” and “moderately satisfactory,” and dissatisfaction was defined when the patient answered “unsatisfactory” and “poor”. Postoperative complications and patients who eventually underwent THA or secondary hip-preserving surgery were also recorded.

### Statistical analyses

The SPSS version 23.0 statistical software was used for all data statistics (IBM Cooperation, USA). For intragroup analysis, a two-sided paired *t* test was used to analyze the changes in normally distributed HHS and VAS score from before to after surgery, while the Mann-Whitney *U* test was used for intergroup analyses. The survival rate was calculated using the Kaplan-Meier method, and we defined the end point as revision to THA. Survival subgroups were divided according to ARCO stage and etiology. We calculated hazard ratios (HRs) and 95% confidence interval (CI) using Cox hazard proportional model to assess the HR of conversion to THA for patients undergoing autologous or allogeneic fibular grafts. Three multivariate models were used by controlling categorical covariates, including age, BMI, ARCR stage, and etiologies. Most of the abovementioned indexes were presented as mean ± standard deviation (SD) with or without range values. Patient satisfaction was shown as a percentage. All statistical analyses were considered significant when the *p* value was < 0.05.

## Results

In the study, 117 patients (153 hips) were finally included. The baseline characteristics of the patients are shown in Table 1. The mean operation time was significantly longer (102.3 ± 18.3 min vs 83 ± 19.5 min, *p* < 0.05) and the mean cost of surgical materials was significantly lower (1080 ± 1587.1 renminbi vs 13707.8 ± 3779.5 renminbi, *p* < 0.05) in group A than in group B. However, the mean amount of bleeding was significantly greater in group A (169 ± 78 ml vs 129.5 ± 67.2 ml, respectively, *p* < 0.05).

The clinical outcomes are presented in Table 2. The preoperative HHS in the 2 groups were both significantly increased after surgery (*p* < 0.05) and preoperative VAS were both significantly decreased after surgery (*p* < 0.05), but no significant differences in the HHS and VAS score at the last follow-up were found between the 2 groups (*p* > 0.05). As for the FJS of the hips at the last follow-up, no significant difference was found between groups A and B (58.1 ± 24.8 vs 60.9 ± 23.3, respectively, *p* > 0.05), which meant that the level of forgetting the joint was similar. The patient satisfaction rates were 86% in group A and 84.5% in group B.

**Table 2:** Summary of clinical assessment

Parameters	Group A		Group B		*p* value for intergroup
	Mean ± SD	*p* value for intragroup	Mean ± SD	*p* value for intragroup	
Pre-HHS	65 ± 7.2	< 0.001	66 ± 5.9	< 0.001	0.3642
Post-HHS	80.3 ± 14.5		82.4 ± 13.6		0.3821
Pre-VAS	6.3 ± 1.1	< 0.001	6.1 ± 1	< 0.001	0.2633
Post-VAS (last follow-up)	2.3 ± 1.6		2.2 ± 2.2		0.7749
Post-hip FJS	58.1 ± 24.8		60.9 ± 23.3		0.4959
Patient satisfaction (%)	86%		84.5%		0.803

Note: *HHS* Harris Hip Score, *VAS* visual analog scale, *FJS* forgotten joint score, *SD* standard deviation, *N/A* not available

**Fig. 2:**
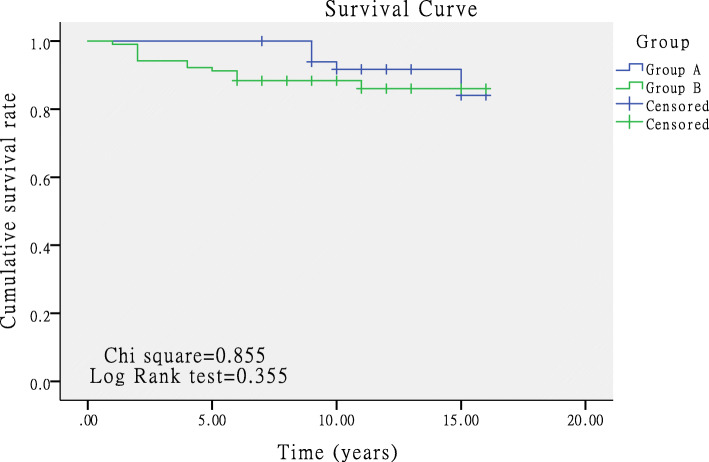
Kaplan-Meier survival curves at 15 years with conversion to THA as the end point between groups A and B (*p* = 0.355)

The survival rates of 117 patients (153 hips) were calculated by using K-M method. The survival rate at 5 years was better in group A than in group B (100% VS 91.2%, *p* = 0.032), while the survival analysis results showed no significant difference in 10-year and 15-year survival rate between groups A and B (*p* = 0.35 and 0.355, respectively; Fig. 2). The 10- and 15-year survival rates were respectively 91.7% and 84.1% in group A, and 88.3% and 86% in group B.

In group A, 6 hips with alcoholic ONFH (12%, 6/50) were converted to THA at a mean follow-up of 11.2 years postoperatively, including 2 hips in ARCO stage IIB, 2 in IIC, 1 in IIIB, and 1 in IIIC. In group B, 13 hips (12.6%, 13/103) were treated with THA at a mean follow-up of 4.1 years postoperatively, including 2 hips in ARCO stage IIB, 6 in IIC, 1 in IIIA, and 4 in IIIC. In addition, 2 hips with steroid-induced ONFH (1.9%, 2/103), which were in the ARCO IIB and IIC stages, respectively, undergone a acetabular rim and femoral osteochondroplasty due to the limited joint movements with only mild pain at 3 and 7 years postoperatively, and they did not convert to THA at the last follow-up. Details are shown in Table 3.

**Table 3:** Summary of survival rate

Parameters	Group A	Group B	*p* value
Hips converting to THA	6(12%)	13(12.4%)	
Hips converting to femoral head-acetabularplasty	0	2(1.9%)	
Time converting to THA (years; mean)	11.2	4.1	
Time without converting to THA (years; mean)	13.1	10	
Survival rate at 5 years	100%	91.2%	0.032
Survival rate at 10 years	91.7%	88.3%	0.35
Survival rate at 15 years	84.1%	86%	0.355

Note: *THA* total hip arthroplasty

The HRs for risk factors of conversion to THA for patients in group A and group B are presented in Table 4. There were no differences in the HRs of conversion to THA for patients undergoing autologous or allogeneic fibular grafts in univariate analysis as well as in multivariate analysis (*p* > 0.05). In the multivariate Cox model, the HR of conversion to THA was significantly increased in patients who were more than 37 years old (HR = 6.931, 95%CI = 1.996–24.074), while no differences were found in other categorical covariates.

**Table 4:** The hazard ratios for risk factors of conversion to THA for patients undergoing autologous or allogeneic fibular grafts

Variables	Model 1	Model 2	Model 3	Model 4
	HR (95% CI)	HR (95% CI)	HR (95% CI)	HR (95% CI)
Surgical methods				
Autologous fibular grafts	1.00 (reference)	1.00 (reference)	1.00 (reference)	1.00 (reference)
Allogeneic fibular grafts	1.624 (0.583–4.526)^†^	1.77 (0.634–4.936)^†^	1.897 (0.69–5.218)^†^	1.716 (0.615–4.786)^†^
Age				
≤ 37 years		1.00 (reference)		
> 37 years		6.931 (1.996–24.074)*		
BMI				
≤ 24		1.00 (reference)		
> 24		2.249 (0.841–6.015)^†^		
ARCR stage				
Stage II		1.00 (reference)		
Stage III		0.823 (0.312–2.173)^†^		
Etiologies				
Glucocorticoid			0.881 (0.16–4.844)^†^	
Alcoholic			2.817 (0.623–12.739)^†^	
Traumatic				0.395 (0.052–3.003)^†^
Idiopathic				0.905 (0.119–6.891)^†^

Note:

Model 1: Cox hazard proportional analysis without adjustment

Model 2: Multivariate Cox hazard proportional analysis including age, BMI, and ARCR stage

Model 3: Multivariate Cox hazard proportional analysis including glucocorticoid and alcoholic

Model 4: Multivariate Cox hazard proportional analysis including traumatic and idiopathic

*p < 0.05, ^†^ > 0.05

In group A, 3 patients were found to have weakened strength in hallux plantar flexion and dorsiflexion on the operative side, which was considered as indicative of a common peroneal nerve injury. Eventually, 3 months after surgery, 2 patients recovered, but the other patient had not. In group B, one incision sustained exudation and delayed union until 3 weeks after operation. A total of 31 patients had a postoperative fever due to immunologic rejection, with a mean duration of 2.6 days, so we extended the use of antibiotics to prevent infection while using a low-dose methylprednisolone sodium succinate to prevent inflammation in these cases. Pulmonary embolism occurred in 1 patient, who recovered after treatment. In the last follow-up, in group A, 5 patients had donor numbness without pain. The percentage of asymptomatic donor sites was 90%. Details are shown in Table 5.

**Table 5:** Summary of complications

Complications	Group A	Group B
Common peroneal nerve injury	3(6%)	0
Incision exudation and delayed union	0	1(1%)
Postoperative fever due to immunologic rejection	0	31(30.1%)
Pulmonary embolism	0	1(1%)
Numbness and pain in the donor sites	5(10%)	N/A

*N/A* not available

## Discussion

At present, the treatment of ONFH in young and middle-aged patients is still controversial. Early diagnosis is of great importance [30], and early surgical intervention is effective for delaying the progress of necrosis and osteoarthritis [31, 32]. Many surgeons are willing to perform hip-preserving surgeries for young and middle-aged patients with ONFH. However, no consensus has been reached on the effectiveness of so many hip-preserving surgeries [9, 33]. The fibular grafting method used in this study is also one of the hot topics in this field of study [20].

During the period of necrosis and repair (ARCO stages II and III), stress could be concentrated between the necrotic and newly formed bones under the load-bearing condition, which would lead to a mild fracture of the bone trabecula, and affect the mechanical properties of the bone structure and repair of the necrotic area. Finally, the necrotic bone in the load-bearing area would collapse when the subchondral bone breaks. In view of the treatment of patients in these 2 stages, the following 4 problems must be solved to repair the necrotic area [34]: (1) improvement of blood flow in the femoral head and promotion of regeneration of blood vessels; (2) effective removal of the necrotic bone; (3) reconstruction of the cartilage in the collapsed area of the femoral head to restore its shape and improve the matching relationship between the femoral head and the acetabulum; and (4) finally, improvement of the mechanical properties of the femoral head and prevention of its collapse.

CD combined with autologous or allogeneic fibular grafting can meet the 4 conditions well. The columnar supporting material, first proposed by Phemister in 1949 for the treatment of ONFH [27], was found to increase the rate of transformation of the structure into living bone and decrease the incidence of collapse. Some surgeons preferred autologous fibular grafting, while others were willing to perform allogeneic fibular grafting [20, 25, 26, 35].

Recently, supporting materials mainly included 3 types, namely vascularized fibula, non-vascularized fibula, and tantalum rod. Vascularized fibular grafting requires a free peroneal artery and anastomosis with the lateral femoral circumflex artery [7, 23]. This surgery can increase the blood supply in the femoral head, but the accompanying trauma is severe. Furthermore, the technical requirements are also high, and vascular embolism may even occur among a few patients in a short period after operation, which may conversely have a negative effect on creeping substitution [36]. The tantalum rod is a porous tantalum metal with an elastic modulus similar to the fibula. However, in recent years, it was found that the host bone was unable to grow in the implanted titanium rod, resulting in further collapse of the femoral head [17, 37]. In addition, the subsequent THA was more difficult. Ma et al. [17] reported that only 55 hips (52.9%) survived after porous tantalum rod implantation in the mean follow-up of 42 months. After retrieving the tantalum rod and femoral head for pathological and electron microscopic observation, Tanzer et al. [37] found that the failure of the tantalum rod implantation was related to minimal bone ingrowth. Therefore, the use of tantalum rods was also gradually reduced, and fibular grafting become the mainstream treatment [20, 38]. Nevertheless, the choice between autogenous and allogeneic fibular grafting has been controversial in academia.

In this study, we performed CD combination with autologous or allogeneic fibular grafting. On the one hand, CD can reduce the intraosseous pressure, stimulate the revascularization of the femoral head, and reconstruct the intraosseous circulation [39, 40]. On the other hand, non-vascularized autologous and allogeneic fibular grafting can improve the mechanical properties [21, 22, 27]. The elimination of necrotic bone should be effective to decrease its distribution and accelerate new bone growth [41].

Not only were the postoperative HHS greatly improved when compared with the preoperative levels between the 2 surgical methods (group A: from 65 ± 7.2 to 80.3 ± 14.5, *p* < 0.05, group B: from 66 ± 5.9 to 82.4 ± 13.6, *p* < 0.05), but also the survival rates of patients without conversion surgery to THA were satisfactory at a mean postoperative follow-up of 13.1 years in group A and 10 years in group B (Tables 2 and 3). In addition, the Kaplan-Meier analysis also revealed the high survival rate in groups A and B, indicating their great effect on the delaying the conversion to THA. Zeng et al. [26] retrospectively reviewed 18 patients with non-traumatic bilateral ONFH who underwent non-vascularized allogeneic fibular graft in one hip and, concurrently, one-stage THA on the contralateral side. They found that the overall survival rate of non-vascularized fibular allografting was 77.8% at a mean follow-up period of 53.3 months. Surprisingly, although the overall survival rates were similar, the average time of conversion to THA in group B was 4.1 years compared to 11.2 years in group A, which may result from the immunologic rejection and influence the repairing environment at an early stage in group B [32]. The patients needed revision to THA because of the collapse of femoral head and subsequent development of osteoarthritis. As for the autologous fibular graft, most studies focused on vascularized grafts for their reliable curative effect. Kawate et al. [25] reported that the overall survival rates (71 hips) could reach 83% at a mean follow-up of 7 years after free vascularized fibular grafting for the treatment of ONFH. They recommended that the degree of osteonecrosis should be less than 300° of the femoral head. Some studies reported that the survival rate with vascularized fibular grafting were higher than that with non-vascularized autologous fibular grafting, while others reported no significant difference between the two methods. Plakseychuk et al. [21] reported 86% survivorship in stage I and II ONFH after treatment with vascularized fibular grafting and only 30% survivorship after non-vascularized fibular grafting at the mean time of 7 years. However, in a study on large osteonecrotic lesions of the femoral head by Kim et al. [22], no significant difference was found in the 3-year survival rate between vascularized and non-vascularized fibular grafts (*p* > 0.05). Tetik et al. [42] demonstrated that although the clinical outcomes of vascularized fibular grafting were better than that of non-vascularized fibular grafting during the 1-year follow-up, no significant radiological difference was found between two procedures. Surprisingly, in spite of ARCO stage II or III, survival rate of 5-year follow-up was 100% and 10-year follow-up was 91.7% in group A. This rate of success in the early and mid-term has rarely been reported in other studies, ranging from 30 to 92.6% [21, 22, 26, 32, 38]. Keizer et al [32] performed the non-vascularized fibular allografts for 60 hips, showing a clinical survival rate of 49% at 6 years and 38% at 10 years. In our opinion, the explanation of the results of high survival rates might be the following 3 surgical techniques: (1) Enough cancellous bone was grafted layer by layer and tightly impacted in all directions. (2) We supposed that the main effect of fibular graft on was its mechanical support but not the vascular implant, which was consistent with the results. (3) After surgery, all patients were demanded for non-weight bearing within the first 6 weeks and total weight bearing until the sixth postoperative month so that there was enough time for bone necrotic area to repair.

Compared with the allogeneic fibular graft, the autogenous fibular graft can better avoid immunologic rejection and save more medical resources and reduce surgical costs, but its use are constrained by the limited materials, longer operation time, and potential donor site complications. During operation, more attention should be paid to preserving the common fibular nerve. Although the allogeneic fibula was derived from the allogeneic bone and may cause immunologic rejection, its immunogenicity could be greatly reduced by bacterial inactivation, marrow removal, quick freezing, etc. [43]. Postoperative immune rejection symptoms can disappear in a short term in conjunction with intravenous administration of a low-dose glucocorticoid [44]. In group B, 2 patients needed additional acetabular rim and femoral osteochondroplasty. This is a surgery for treating the femoroacetabular impingement when the ONFH progressed [45]. During the surgery, we removed the abnormal bony structure of the femoral head and acetabular rim to improve the range of motion, which can delay THA again. There were no relative reports comparing this procedure in the two groups. According to the multivariate Cox model, we found no differences between HR of conversion to THA and etiologies. Yoo et al. [46] also found survival rates were associated with the patient’s age but not etiology and stages of ONFH.

**Fig. 3:**
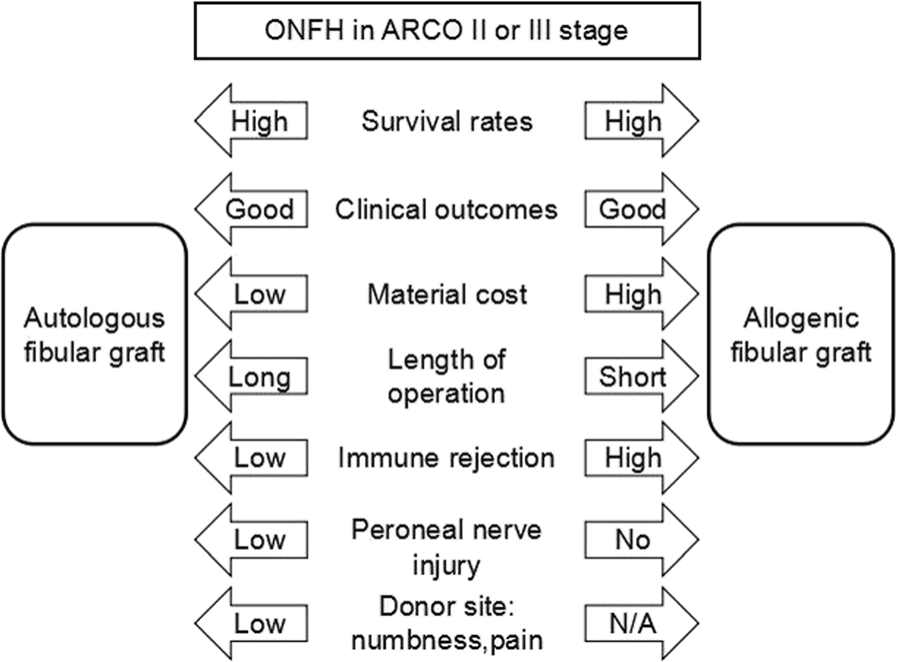
A photograph of important factors for considering autologous or allogeneic fibular graft young and middle-aged patients with ONFH in ARCO II or III stage

In summary, whether performing the autologous or allogeneic fibular grafting for young and middle-aged patients with ONFH of ARCO stage II or III is still a challenge for orthopedic surgeons because these patients present a highly heterogeneous group with extensively different symptoms, complaints, and expectations. Therefore, to achieve high postoperative satisfaction, a differentiated approach should be considered according to survival rates, clinical outcomes, bearable range of economic capability for patients, material cost, length of operation, postoperative immune rejection symptoms, and donor site complications (Fig. 3).

### Limitations

Accompanied by evident strengths, several limitations exist in this study. First, prospective studies were currently in demand, but this was a retrospective comparative study. However, prospective data for long-term follow-up are difficult to collect in a short time. Second, the follow-up time in group A was significantly longer than that in group B, which might have made a difference in the outcome, but error in the calculation of survival rates could be avoided by using the Kaplan-Meier method. Another limitation was the small sample size, especially in group A, which may result in significant difference in age. Although autologous and allogeneic techniques had been performed since 2003, all the patients who met the indication only underwent allogeneic fibular grafting after 2007 owing to its excellent outcome, less invasiveness, and less complications, which were our early clinical experiences. Simultaneously, medical records before 2002 were incomplete. Finally, we focused on the clinical survival rates but not on the radiographic survival rates because some patients did not undergo radiography regularly in our outpatient clinic. To draw a more reliable conclusion, a randomized controlled trial with larger samples is needed in further study.

## Conclusions

In this study, we found that more than 37 years old will increase the ratio of failure. During the long-term follow-up periods, CD and impaction grafting combined with autologous or allogeneic fibular grafting could achieve good clinical outcomes, which greatly delayed the time for converting to THA, and their long-term therapeutic effects and survival rates were similar, which were worthy of application and promotion.

## Supplementary Information

**Additional file 1.** Ethical Committee Approval.

## Data Availability

All data generated or analyzed during this study are included in this published article. The datasets generated during and/or analyzed during the current study are also available from the corresponding author on reasonable request.

## Abbreviations

ONFH: Osteonecrosis of the femoral head
THA: Total hip arthroplasty
ARCO: Association research circulation osseous
HHS: Harris Hip Score VAS Visual analog scale
FJS: Forgotten joint score
CD: Core decompression
HRs: Hazard ratios
CI: Confidence interval
SD: Standard deviation

## References

[1] Petek D, Hannouche D, Suva D (2019). Osteonecrosis of the femoral head: pathophysiology and current concepts of treatment. EFORT Open Rev..

[2] Fukushima W, Fujioka M, Kubo T, Tamakoshi A, Nagai M, Hirota Y (2010). Nationwide epidemiologic survey of idiopathic osteonecrosis of the femoral head. Clin Orthop Relat Res..

[3] Mont MA, Salem HS, Piuzzi NS, Goodman SB, Jones LC (2020). Nontraumatic osteonecrosis of the femoral head: where do we stand today?: A 5-Year Update. J Bone Joint Surg Am..

[4] Rajaee SS, Campbell JC, Mirocha J, Paiement GD (2018). Increasing burden of total hip arthroplasty revisions in patients between 45 and 64 years of age. J Bone Joint Surg Am..

[5] Kahlenberg CA, Swarup I, Krell EC, Heinz N, Figgie MP (2019). Causes of revision in young patients undergoing total hip arthroplasty. J Arthroplasty..

[6] Zeng Y, He S, Feng W, Li F, Li J, Jian L (2013). Vascularised greater trochanter bone graft, combined free iliac flap and impaction bone grafting for osteonecrosis of the femoral head. Int Orthop..

[7] Ünal MB, Cansu E, Parmaksızoğlu F, Cift H, Gürcan S (2016). Treatment of osteonecrosis of the femoral head with free vascularized fibular grafting: results of 7.6-year follow-up. Acta Orthop Traumatol Turc..

[8] Feng WJ, Ye PC, Ni SH, Deng P, Lu L, Chen JL (2019). One-stage simultaneous hip-preserving surgeries for the management of bilateral femoral head osteonecrosis: a mean 7.0-year follow-up. J Orthop Surg Res.

[9] Tripathy SK, Goyal T, Sen RK (2015). Management of femoral head osteonecrosis: current concepts. Indian J Orthop..

[10] Wang J, Wang J, Zhang K, Wang Y, Bao X (2018). Bayesian network meta-analysis of the effectiveness of various interventions for nontraumatic osteonecrosis of the femoral head. Biomed Res Int..

[11] Yue J, Guo X, Wang R, Li B, Sun Q, Liu W (2020). Single approach to double-channel core decompression and bone grafting with structural bone support for treating osteonecrosis of the femoral head in different stages. J Orthop Surg Res..

[12] Migliorini F, Maffulli N, Eschweiler J, Tingart M, Baroncini A. Core decompression isolated or combined with bone marrow-derived cell therapies for femoral head osteonecrosis. Expert Opin Biol Ther. 2020;30:1–8. 10.1080/14712598.2021.1862790 33297783

[13] Nazal MR, Parsa A, Martin SD (2019). Mid-term outcomes of arthroscopic-assisted core decompression of precollapse osteonecrosis of femoral head-minimum of 5 year follow-up. BMC Musculoskelet Disord..

[14] Sadile F, Bernasconi A, Russo S, Maffulli N (2016). Core decompression versus other joint preserving treatments for osteonecrosis of the femoral head: a meta-analysis. Br Med Bull..

[15] Floerkemeier T, Lutz A, Nackenhorst U, Thorey F, Waizy H, Windhagen H (2011). Core decompression and osteonecrosis intervention rod in osteonecrosis of the femoral head: clinical outcome and finite element analysis. Int Orthop..

[16] Floerkemeier T, Thorey F, Daentzer D, Lerch M, Klages P, Windhagen H (2011). Clinical and radiological outcome of the treatment of osteonecrosis of the femoral head using the osteonecrosis intervention implant. Int Orthop..

[17] Ma J, Sun W, Gao F, Guo W, Wang Y, Li Z (2016). Porous tantalum implant in treating osteonecrosis of the femoral head: still a viable option?. Sci Rep..

[18] Kawano K, Motomura G, Ikemura S, Kubo Y, Fukushi J, Hamai S (2020). Long-term hip survival and factors influencing patient-reported outcomes after transtrochanteric anterior rotational osteotomy for osteonecrosis of the femoral head: A minimum 10-year follow-up case series. Mod Rheumatol..

[19] Sugioka Y, Hotokebuchi T, Tsutsui H. Transtrochanteric anterior rotational osteotomy for idiopathic and steroid-induced necrosis of the femoral head. Indications and long-term results. Clin Orthop Relat Res. 1992;(277):111–20. 1555330

[20] Korompilias AV, Beris AE, Lykissas MG, Kostas-Agnantis IP, Soucacos PN (2011). Femoral head osteonecrosis: why choose free vascularized fibula grafting. Microsurgery..

[21] Plakseychuk AY, Kim SY, Park BC, Varitimidis SE, Rubash HE, Sotereanos DG (2003). Vascularized compared with nonvascularized fibular grafting for the treatment of osteonecrosis of the femoral head. J Bone Joint Surg Am..

[22] Kim SY, Kim YG, Kim PT, Ihn JC, Cho BC, Koo KH (2005). Vascularized compared with nonvascularized fibular grafts for large osteonecrotic lesions of the femoral head. J Bone Joint Surg Am..

[23] Aldridge JM, Berend KR, Gunneson EE, Urbaniak JR (2004). Free vascularized fibular grafting for the treatment of postcollapse osteonecrosis of the femoral head. Surgical technique. J Bone Joint Surg Am.

[24] Eward WC, Rineer CA, Urbaniak JR, Richard MJ, Ruch DS (2012). The vascularized fibular graft in precollapse osteonecrosis: is long-term hip preservation possible?. Clin Orthop Relat Res..

[25] Kawate K, Yajima H, Sugimoto K, Ono H, Ohmura T, Kobata Y (2007). Indications for free vascularized fibular grafting for the treatment of osteonecrosis of the femoral head. BMC Musculoskelet Disord..

[26] Zeng Y, Qi X, Feng W, Li J, Li F, Zeng J (2015). One-sided hip-preserving and concurrent contralateral total hip arthroplasty for the treatment of bilateral osteonecrosis of the femoral head in different stages: short-medium term outcomes. BMC Musculoskelet Disord..

[27] Phemister DB (1949). Treatment of the necrotic head of the femur in adults. J Bone Joint Surg Am..

[28] Penix AR, Cook SD, Skinner HB, Weinstein AM, Haddad RJ (1983). Femoral head stresses following cortical bone grafting for aseptic necrosis. A finite element study. Clin Orthop Relat Res.

[29] Behrend H, Giesinger K, Giesinger JM, Kuster MS (2012). The “forgotten joint” as the ultimate goal in joint arthroplasty: validation of a new patient-reported outcome measure. J Arthroplasty..

[30] Larson E, Jones LC, Goodman SB, Koo KH, Cui Q (2018). Early-stage osteonecrosis of the femoral head: where are we and where are we going in year 2018?. Int Orthop..

[31] Fang T, Zhang EW, Sailes FC, McGuire RA, Lineaweaver WC, Zhang F (2013). Vascularized fibular grafts in patients with avascular necrosis of femoral head: a systematic review and meta-analysis. Arch Orthop Trauma Surg..

[32] Keizer SB, Kock NB, Dijkstra PD, Taminiau AH, Nelissen RG (2006). Treatment of avascular necrosis of the hip by a non-vascularised cortical graft. J Bone Joint Surg Br..

[33] Xie H, Wang B, Tian S, Liu B, Qin K, Zhao D (2019). Retrospective long-term follow-up survival analysis of the management of osteonecrosis of the femoral head with pedicled vascularized iliac bone graft transfer. J Arthroplasty..

[34] Babis GC, Sakellariou V, Parvizi J, Soucacos P (2011). Osteonecrosis of the femoral head. Orthopedics..

[35] Lakshminarayana S, Dhammi IK, Jain AK, Bhayana H, Kumar S, Anshuman R (2019). Outcomes of core decompression with or without nonvascularized fibular grafting in avascular necrosis of femoral head: short term follow-up study. Indian J Orthop..

[36] Meloni MC, Hoedemaeker WR, Fornasier V (2016). Failed vascularized fibular graft in treatment of osteonecrosis of the femoral head. A histopathological analysis. Joints..

[37] Tanzer M, Bobyn JD, Krygier JJ, Karabasz D (2008). Histopathologic retrieval analysis of clinically failed porous tantalum osteonecrosis implants. J Bone Joint Surg Am..

[38] He W, Li Y, Zhang Q, Wang H, Fang B, Pang Z (2009). Primary outcome of impacting bone graft and fibular autograft or allograft in treating osteonecrosis of femoral head. Zhongguo Xiu Fu Chong Jian Wai Ke Za Zhi..

[39] Rajagopal M, Samora JB, Ellis TJ (2012). Efficacy of core decompression as treatment for osteonecrosis of the hip: a systematic review. Hip Int..

[40] Marker DR, Seyler TM, Ulrich SD, Srivastava S, Mont MA (2008). Do modern techniques improve core decompression outcomes for hip osteonecrosis?. Clin Orthop Relat Res..

[41] Lieberman JR (2004). Core decompression for osteonecrosis of the hip. Clin Orthop Relat Res..

[42] Tetik C, Başar H, Bezer M, Erol B, Ağir I, Esemenli T (2011). Comparison of early results of vascularized and non-vascularized fibular grafting in the treatment of osteonecrosis of the femoral head. Acta Orthop Traumatol Turc..

[43] Yu HB, Shen GF, Wei FC (2007). Effect of cryopreservation on the immunogenicity of osteoblasts. Transplant Proc..

[44] Moraschini V, Almeida DCFD, Calasans-Maia MD, Kischinhevsky ICC, Louro RS, Granjeiro JM (2020). Immunological response of allogeneic bone grafting: A systematic review of prospective studies. J Oral Pathol Med..

[45] Espinosa N, Beck M, Rothenfluh DA, Ganz R, Leunig M (2007). Treatment of femoro-acetabular impingement: preliminary results of labral refixation. Surgical technique. J Bone Joint Surg Am.

[46] Yoo MC, Kim KI, Hahn CS, Parvizi J (2008). Long-term follow up of vascularized fibular grafting for femoral head necrosis. Clin Orthop Relat Res..

