# Supplementary material for: Long-term survival and clinical outcomes of non-vascularized autologous and allogeneic fibular grafts are comparable for treating osteonecrosis of the femoral head
Source: J Orthop Surg Res. 2021 Feb 4;16:109. doi: 10.1186/s13018-021-02246-3 (PMC7863426; doi:10.1186/s13018-021-02246-3)
Supplement: Supplementary file 1 — Additional file 1. Ethical Committee Approval. [file 13018_2021_2246_MOESM1_ESM.pdf]

伦理审查意见

Review Opinions

意见号 NO. K【2019】122

项目名称<br>Protocol Title	髓心减压联合自体腓骨支撑或异体腓骨支撑治疗股骨头坏死：一项回顾性的中远期随访病例研究		
试验目的<br>Objective	□Ⅰ期临床试验；□Ⅱ期临床试验；□Ⅲ期临床试验；□Ⅳ期临床试验；□生物等效性试验；□进口药注册临床试验；□上市药再评价；□中药保护品种试验；■科研课题；□医疗器械临床试用；□医疗器械临床验证；□研究生教育；□其他		
审查类别<br>Review Category	■初始审查<br>□跟踪审查（□修正案审查 □年度/定期研究进展审查 □违背方案审查 □SAE 审查 □暂停/终止研究审查 □研究完成审查 □其他________）<br>□复审		
科室/专业名称<br>Specialties	三骨科	主要研究者<br>Principal Investigator	曾意荣 主任医师
审查材料<br>documents	1、伦理审查申请；<br>2、临床研究方案（版本号 1.0）；<br>3、知情同意书（版本号 1.0）；<br>4、主要研究者专业履历；<br>5、主要研究者及研究团队利益冲突声明；<br>6、病例报告表（版本号 1.0）。	申办单位<br>Sponsor	本院三骨科
审查方式<br>Review modes	■快速审查 Expedited<br>□会议审查 Full Board，会议时间 Date: （另附会议签到副本）		
审查结论<br>Decision	□同意<br>approve<br>■作必要的修正后同意<br>Minor Revision.<br>□作必要的修正后重审<br>Major Revision<br>□不同意<br>Disapprove<br>□终止或暂停已批准的研究<br>Termination or Suspension<br>□转伦理会议审查<br>Transfer to Full Board Review	年度/定期跟踪审查频率<br>Annual Follow-Up Review Frequency	12 个月

认证资质： 中医药研究伦理审查体系认证（CAP）

审查意见<br>Opinions	根据卫计委《涉及人的生物医学研究伦理审查办法》（2016 年）、SFDA《药物临床试验伦理审查工作指导原则》（2010 年）、《医疗器械临床试验质量管理规范》（2016 年），以及 WMA《赫尔辛基宣言》和 CIOMS《人体生物医学研究国际道德指南》的伦理原则，经本伦理委员会审查，修订意见如下：<br>1、研究方案：收集“2004.1~2011.12”期间的患者病例数据，观察点不涉及门诊或复查，请明确如何获取患者知情同意？如无法获取，建议作知情同意例外的申请并提交保密协议。<br>请于 2 个月内将复审申请表、伦理审查意见复印件、修改说明（请于院内网伦理专栏-资料下载-流程 2 下载模板）及修改后的文件（修改后的版本，需更新版本号/版本日期）、补充文件（如有）共一式 2 份报伦理委员会复审。若对审查意见有不同观点，请附加相关“说明报告”。
联系方式（委员/秘书）<br>Contact/EC Secretary	黎老师，外线：020-36588667（周一、周三，14:30-17:30）/020-36591965；医院内线：1530（周一、周三，14:30-17:30）/1965；传真 020-36591346；邮箱：gztcmlunli@163.com
主任委员/被授权者签名<br>Signature of the Chair/ Authorized Person	日期： 2019-11-6

申明：本伦理委员会严格按照中国 GCP 及相关法规组成和工作。　广州中医药大学第一附属医院伦理委员会（盖章）
